# The protocol for validating phone interview tools on post-discharge consequences of road traffic injuries

**DOI:** 10.5249/jivr.v12i3.1368

**Published:** 2020-08

**Authors:** Soudabeh Marin, Homayoun Sadeghi-Bazargani, Mostafa Farahbakhsh, Alireza Ala, Hossein Poustchi, Faramarz Pourasghar

**Affiliations:** ^*a*^ Department of Biostatistics and Epidemiology, School of Health, Tabriz University of Medical Sciences, Tabriz, Iran.; ^*b*^ Road Traffic Injury Research Center, Tabriz University of Medical Sciences, Tabriz, Iran.; ^*c*^ Research Center of Psychiatry and Behavioral Sciences, Tabriz University of Medical Sciences, Tabriz, Iran.; ^*d*^ Department Emergency Medicine, Emergency Medicine Research Team, Tabriz University of Medical Sciences, Tabriz, Iran.; ^*e*^ Liver and Pancreatobiliary Disease Research Institute, Shariati Hospital, Tehran University of Medical Sciences, Tehran, Iran.

**Keywords:** Road Traffic Injury, Post-discharge, Post-crash, Persian Traffic Cohort, Registry, Phone Survey

## Abstract

**Background:**

Road Traffic Injuries (RTIs) impose a worldwide burden on public health and economy. RTIs result in a wide range of immediate and long-term consequences. However, little is known about post-discharge consequences of RTIs at national levels. In addition, reliable and producing valid data mostly through prospective studies is fundamentally required to address the issue. The aim of this paper was to describe the research protocol for development and psychometric evaluation of post-discharge consequences of road traffic injuries as part of the Persian Traffic Cohort (PTC) and complementary to the Iranian Integrated Road Traffic Injury Registry (IRTIR).

**Methods:**

Literature review and expert’s opinion were used to develop data collection tools. Registry timeframe was designed based on experts’ opinion. Reliability of tools will be assessed using intra- and inter-rater reliability. The pilot phase of the Phone interviews on Post-discharge Consequences of Road Traffic Injuries (PCRTI) will be conducted in Tabriz in 2019.

**Results:**

The PCRTI is designed to be applied at three different time points: one, six and twelve months after the crash. The PCRTI tools’ main domains are: demographic, psychological, medical, social and financial which will be assessed through PC-PTSD, PHQ2, WHODAS, SES-Iran, EQ-5D-3L and Economic assessment standardized tools. The descriptive outcomes will be reported to the Ministry of Health and Medical Education of Iran.

**Conclusions:**

The protocol satisfies the requirements of developing valid data collection tools for PCRTI.

## Introduction

Road Traffic Injuries (RTIs) are a global public health problem and major leading cause of death and disability around the world.^1^ RTIs result in a wide range of consequences from immediate outcomes such as mortality and disability to long-term psycho-social and socioeconomic consequences.^2-5^ According to World Health Organization (WHO) reports, every year, over 1.2 million people die due RTIs around the world and about 50 million endure non-fatal injuries. Moreover, the impact of RTIs on economic are estimated to be between 1% to 3% of the counties’ gross national product.^6^ Studies showed that the quality of life (QoL) of road traffic victims significantly decrease following the crash and majority of them never reach the general population standards of QoL.^7^ RTIs are major portion of injuries and the third leading cause of death in Iran. Years of healthy life lost due to RTIs estimated to be 1.2 million years.^8^ A study in Iran estimated the cost of road traffic injuries and death to be 6.46% of gross national income.^9^ Nevertheless, the real cost of road traffic crashes in Iran seems to be much higher than the stated figure. It should also include cost to the roadway infrastructure as well as to the vehicle itself. Costs related to lost-income in death, hospitalization or physical disability are estimated 1.4 billion US dollar. ^10^ However, researches on road traffic crashes (RTCs) predominantly focused on immediate and severe outcomes, a large number of road traffic victims suffer from permanent disabilities, functional impairments and psychological problems.^4,5,11-14^

Sustainable and significant reduction in RTIs requires reliable and accurate data to inform policy makers, set priority, manage strategies, facilitate cooperation, optimize post-crash services and tack appropriate actions.^1,15^ Trauma registries in high-income countries verified to be beneficial at identifying risk factors and subsequent injury outcomes and determining defects and gaps in care services.^16,17^ However, data sources in Low- and Middle-Income Countries (LMICs), including Iran, are often of poor quality, contain an insufficient number of relevant elements or have difficulty to be assessed which had resulted in weak estimation of morbidity and consequences of road traffic crashes.^8^

Although different organizations or research studies in Iran are gathering road traffic data in Iran, these data mostly limited to crash scene data, pre-hospital and hospital data. Valid and reliable post-crash data, which are fundamentally required for an effective post-crash response,^18^ are scant in Iran. Little is known about post-discharge consequences of road traffic crashes. As the integrated road traffic injury registry is implemented in East Azerbaijan of Iran and the Persian Traffic Cohort is at the same time being implemented in East Azerbaijan capital, there was a profound need for developing valid tools in order to collect data on crash outcomes for post-discharge follow-up of crash victims. As a part of the Persian Traffic Cohort (PTC) and complementary to the Iranian Integrated Road Traffic Injury Registry (IRTIR), the aim of this paper was to describe the research protocol for development and psychometric evaluation of the phone interview measurement tools for post-discharge consequences of road traffic injuries.

**Persian Traffic Cohort**

The Persian Traffic Cohort is the national prospective cohort on road safety and traffic injuries established in Iran to quantify the health effects related with road safety. This cohort is also a parallel contribution to the national population-based study of PERSIAN Cohort. PTC is comprised of two main sections namely Pre-crash Cohort and Post-crash Cohort. 

**Iranian Integrated Road Traffic Injury Registry (IRTIR)**

Iranian Integrated Road Traffic Injury Registry is a comprehensive national road traffic injury registry. Different organizations collaborate in this registry including: Police, Forensic medicine organization, Ministry of Health and Medical Education (MoHME) and Iranian Red Crescent Organization. Each one of these organizations collects road traffic data at a particular station. Then the data will be integrated through an electronic system. IRTIR study was financially supported by Ministry of Health & Medical Education through the contract number 700-1482 95-10-4 between MOH and Tabriz University of Medical Sciences.

**Study protocol**

**Study settings and participants**

The pilot phase of PCRTI project will be conducted in Tabriz in two referral trauma hospitals (Imam Reza and Shohadah university hospitals). Study Participants will be selected from road traffic victims who admitted into trauma hospitals. Baseline road traffic injury data will be extracted from IRTIR databank. Baseline data are being collected through face to face interview at nursing station of integrated road traffic injury registry (IRTIR). The data includes description of the crash, victim’s demographics, crash’s environment data, vehicle-related data and the injured person’s behavior data. Details of development and psychometrics of baseline nursing station data collection tool of IRTIR is provided elsewhere.^19^ The post-discharge registry eligibility criteria will be as follows: being inpatient for at least 24 hours, all types of road traffic crash injuries, all types of road users and having a registered telephone number. The PCRTI will be conducted through phone interviews. Trained coders will explain the study purpose to road traffic victims and will receive informed verbal consent. For children under the age of 18, informed consent will be sought out from their parents or his/her legal guardian. Data will be collected via three separate structured data collection tool packages through telephone interviews. If the victim is unable to answer because of health problems or other disabilities, the most informant family member will be interviewed.

**Developing PCRTI data collection variables and tools**

A comprehensive literature review was done to identify the most important consequences due to road traffic crashes. Main domains and minimum data variables for PCRTI were defined based on literature review and experts’ opinion and the preliminary data collection tool packages were developed. 

**Post-discharge follow-up time-frame**

The timeframe was discussed in a panel of 12 experts who were from different academic backgrounds including epidemiology, medical informatics, medical, psychology, psychiatry, healthcare management, health economics, and forensic medicine. The research group reached a consensus on follow-up timeframe at 1, 6 and 12 months after the crash date.

• One month after the traffic crash (main measurement topics: acute consequences, mortality and psychological consequences.)

• Six months after the traffic crash (main measurement topics: disability and quality of life.)

• Twelve months after the traffic crash (main measurement topics: long lasting consequences, socioeconomic issues.)

The expert panel chose this timeframe because of the following reasons: 1- It is recommended that deaths within 30 days post-crash to be considered as road traffic fatality.^14^ Besides, the first month after the crash and injuries is the period when major health outcomes occurs. 2- According to previous research, most of road traffic victims might be adapted to post-crash condition or their health status have recovered six months after the traffic crash.^20-22^ 3- The influence of RTI on health related quality of life and socioeconomic conditions may not be apparent in short- and medium-term periods. Therefore long-term assessments are also required. Previous studies showed no further improvement in health status after 12 months.^23,24^

**Reliability Assessment**

**Figure 1:**
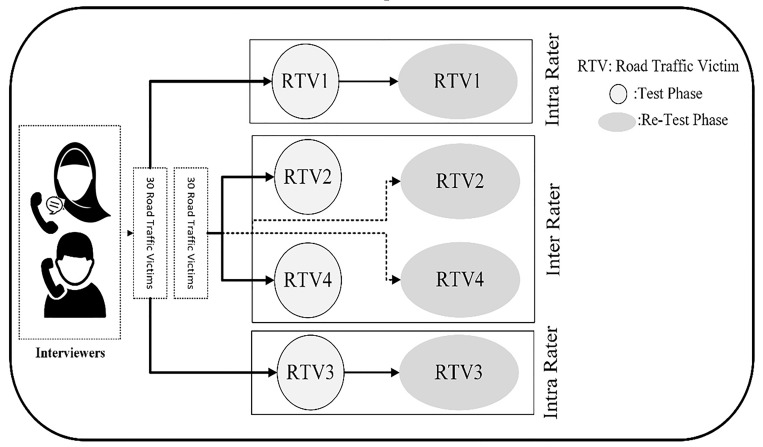
Reliability assessment process for measurement tools of the phone interviews on Post-discharge Consequences of Road Traffic Injuries (PCRTI).

Reliability of data collection tool packages will assess using intra- and inter-rater reliability method. Figure 1 shows PCRTI data collection tool packages’ reliability assessment process. In order to check reliability, two different sample sets of 30 road traffic victims will be selected from IRTIR databank based on the crash dates. One sample set will be used to assess intra-rater reliability and the other will be used to assess inter-rater reliability. Intra-rater reliability will assess using test-retest method. Trained raters will interview with road traffic victims via telephone and fill a structured data collection tool. The victims will be interviewed one week later for the second time by the same raters. In inter-rater reliability assessment phase, re-interview will be done by a different rater. Absolut/mixed Intra-Class Correlation Coefficient (ICC), Kendal tau-b, and Kappa value will be used to assess the reliability. Internal consistency of the tools will assess using Cronbach’s alpha. (Figure 1)

**Data management and reporting**

Data will be entered into IRTIR and PTC electronic linkage database and will be integrated with baseline data. In order to report public health and economic outcomes of RTIs, descriptive and analytical statistics will be reported to the ministry of health and medical education every six month. 

**Data collection tool packages**

**Figure 2:**
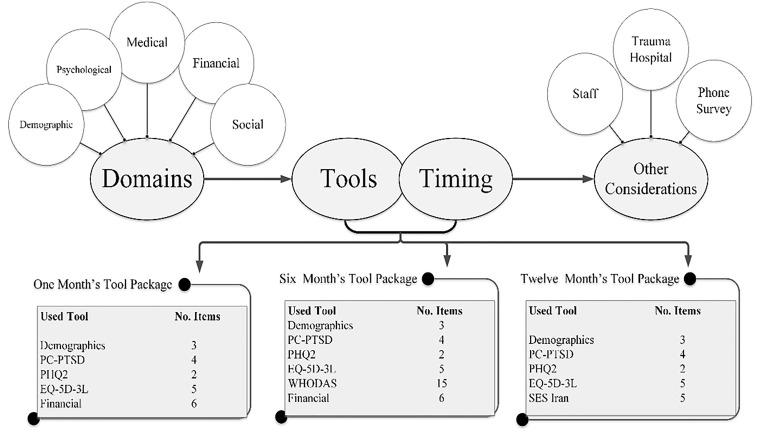
The main domains, time points and contents of PCRTI data collection tool-packages.

Since there were valid and reliable scales in registry’s desired domains, there was no need to develop new tools. Therefore, researchers only designed data collection tool packages suitable for follow-up time points. Three different time points were distinguished to be meaningful for PCRTIs based on experts’ perspectives. The registry time points were as follows:

The main domains, time points and contents of each PCRTI data collection tool-package are shown in Figure 2.

**Measures**

***Post-Traumatic Stress Disorder***

Post-traumatic stress disorder (PTSD) will be assessed using Primary Care Post-Traumatic Stress Disorder (PC-PTSD) which is a 4-item yes/no screening questionnaire designed for use in primary care and other medical settings. The instrument includes an introductory sentence to point respondents to traumatic events.^25^ The PC-PTSD has high diagnostic efficiency. The screening has showed good test-retest reliability (r=80), high sensitivity (0.91%) and specificity (0.80).^26^

***Depression***

The 2-item Patient Health Questionnaire (PHQ2) has been recommended for depression screening in first step approach. PHQ-2 includes the first two items of PHQ-9. The PHQ-2 asks respondents to estimate frequency of major depression and anhedonia symptoms over the past two weeks, scoring each mood from 0 (not at all) to 3 (nearly every day).^27^

***Disability and functioning***

World Health Organization Disability Assessment Schedule (WHODAS) 12-item version was selected to be used in order to assess the overall functioning and disability. The scale covers six life domains including: cognitive, mobility, self-care, interacting with other people, life activities and participation in community activities.^28^ Administration time for 12-item WHO-DAS is less than five minutes.^29^ The full version is considered to be used for yearly face to face interviewed assessments needed in PTC. 

***Health Related Quality of Life***

Health Related Quality of Life (HRQoL) of road traffic victims were assessed using Euro Quality of Life five-dimension three-level (EQ-5D-3L).^30^ The tool covers most of the important domains of disability and has other practical advantages such as brevity, being applicable in all kinds of road traumas as well as being applicable in various age groups and recommended by previous injury-related outcomes study.^31^

***Socio-Economic Status***

Socio-economic status is assessed using ultra short version of socio-economic status tool for health studies in Iran (SES-Iran). The SES ultra-short version was comprised of six items as follows: occupation, years of education, household income per month, financial value of private car and housing.^32,33^

***Economic Impact***

In our study, three cost components including direct medical costs, direct non-medical costs, and indirect costs are estimated to indicate the economic burden of RTIs on victims. Direct costs is assessed through a validated Iranian questionnaire^34,35^ which contained hospitalization, physician visits, diagnostic care (imaging, CT scans, MRI, etc.), medications and rehabilitation as direct medical costs, and travel, accommodation, vehicle damage and any other non- medical expenses resulted by RTIs as direct non-medical costs. The micro-costing approach was used to calculate the direct costs. ^36^ Indirect costs present the lifetime earnings foregone as the result of a RTI; therefore, the amount of lost working days was asked from the patients and then the indirect cost was calculated using the Human Capital Approach.^37^ Assessing the economic Impact of RTIs was applied to 1-monrh and 6-month follow up data collection tools. 

***Employment status***

To reach a better understanding of how RTIs can economically affect the victims’ lives, any changes in their employment status after the crash will be asked through a scale of losing the job, changing the job to another, the same job with lower function, and the same job without any changes. Also the patients were asked to state their purchasing power after the crash as if the costs resulted by RTIs could change it than before or not.

These tools are selected because they are short, simple, and easy to administer, applicable through phone interview, and general interview techniques are sufficient to administer these tools, therefore they are appropriate for the registry purposes.
